# Efficacy and safety of a single switch from etanercept originator to etanercept biosimilar in a cohort of inflammatory arthritis

**DOI:** 10.1038/s41598-020-73183-0

**Published:** 2020-09-30

**Authors:** Maria Chiara Ditto, Simone Parisi, Marta Priora, Silvia Sanna, Clara Lisa Peroni, Angela Laganà, Antonio D’Avolio, Enrico Fusaro

**Affiliations:** 1 Department of General and Specialistic Medicine, Rheumatology Unit, Azienda Ospedaliera Universitaria Città della Salute e della Scienza di Torino, Turin, Italy; 2 Medical Science, Rheumatology Unit, Azienda Ospedaliera Universitaria di Padova, Padua, Italy; 3 Laboratory of Clinical Pharmacology and Pharmacogenetics, Department of Medical Sciences, University of Turin, Amedeo di Savoia Hospital, Turin, Italy

**Keywords:** Biological therapy, Rheumatoid arthritis

## Abstract

AntiTNF-α biosimilars are broadly available for the treatment of inflammatory arthritis. There are a lot of data concerning the maintenance of clinical efficacy after switching from originators to biosimilars; therefore, such a transition is increasingly encouraged both in the US and Europe. However, there are reports about flares and adverse events (AE) as a non-medical switch remains controversial due to ethical and clinical implications (efficacy, safety, tolerability). The aim of our work was to evaluate the disease activity trend after switching from etanercept originator (oETA-Enbrel) to its biosimilar (bETA-SP4/Benepali) in a cohort of patients in Turin, Piedmont, Italy. In this area, the switch to biosimilars is stalwartly encouraged. We switched 87 patients who were in a clinical state of stability from oETA to bETA: 48 patients were affected by Rheumatoid Arthritis (RA),26 by Psoriatic Arthritis (PsA) and 13 by Ankylosing Spondylitis (AS).We evaluated VAS-pain, Global-Health, CRP, number of swollen and tender joints, Disease Activity Score on 28 joints (DAS28) for RA, Disease Activity in Psoriatic Arthritis (DAPSA) for PsA, Health Assessment Questionnaire (HAQ) and Health Assessment Questionnaire for the spondyloarthropathies (HAQ-S),Bath Ankylosing Spondylitis Disease Activity Index (BASDAI) for AS patients. 11/85 patients (12.6%) stopped treatment after switching to biosimilar etanercept. No difference was found between oETA and bETA in terms of efficacy. However, some arthritis flare and AE were reported. Our data regarding maintenance of efficacy and percentage of discontinuation were in line with the existing literature.

## Introduction

Biologics are target-specific, highly effective drugs approved for many pathologic conditions such as inflammatory arthritis, psoriasis, Crohn disease, uveitis, osteoporosis, cancer, HIV, multiple sclerosis and others. In particular, over the last 20 years many advances allowed drugs to actually modify the natural history of rheumatic diseases such as Rheumatoid Arthritis, SpondyloArthritis (including Ankylosing Spondylitis), Psoriatic Arthritis, Reactive Arthritis, and more recently Systemic Lupus Erythematosus, thanks to their effectiveness in reducing disease activity, joint pain, swelling and damage progression. The high cost of Biological Disease Modifying Anti-Rheumatic Drugs (bDMARDs) in the world is the main factor limiting its prescription as a first line of therapy despite the best efficacy and tolerability.

Despite their cost, the three main anti-TNF alpha originators (Humira, Enbrel and Remicade) were amongst the top 20 drugs of the world ranking (which accounted for the total global prescription drug market in the last years); the annual growth for Humira in 2016 (the first product of the ranking) was 15%, accounting for a $16-billion-sale worldwide, which could also be due to its numerous indications^1–8^^.^

Biologics are derived from living cells crossing a complex biotechnological process. The intrinsic nature of these proteins makes it almost impossible to replicate an exact copy (generic) of a biological drug; therefore, biosimilars are products similar to the original drug in the active substance, but not identical for differences in its manufacturing process, including methods used to purify and stabilize cellular lines, which influence post-translational modifications of the proteins (such as glycosylation, etc.)^9–11^.

To date, the patents for 3 anti TNF-alpha (Remicade/Infliximab, Enbrel/Etanercept, Humira/Adalimumab) and one anti B-cells (Mabthera/Rituximab) have expired, thus allowing many biosimilars to be available in the world for the treatment of inflammatory joint diseases.

Several randomized, double-blind, controlled clinical trials versus placebo demonstrated the efficacy and safety of the switch from biologic originators to the biosimilar of infliximab, etanercept and adalimumab as many experiences from trials and real world data are available^12–20^, thus having been approved for the same indications by FDA (including extrapolated suggestions as well)^21^.

However, despite the considerable saving, such shifts to biosimilar drugs are still being debated, principally over their ethical implications. Since the drugs are similar but not identical, the main issues are related to the adverse events and the lack of efficacy, which cannot be excluded. This also implies that biosimilars could theoretically work better than originators, but the variability in effectiveness for a single patient remains an unpredictable datum before effecting the switch.

Despite the fact that extrapolation of indications are debated (especially for the treatment of inflammatory bowel diseases since the mechanisms of action might differ from indications^22^), the use of biosimilars appears to be regulated worldwide by local guidelines if safety and effectiveness are demonstrated in clinical trials for at least one indication. Moreover, data concerning immunogenicity, in at least one clinical trial comparing the development of anti-drug antibodies in patients previously exposed to the originator, are required by regulatory agencies before the approval of the biosimilar^11,23–27^.

A small survey conducted in the UK showed an agreement regarding the switch if the treatment "works as well as my existing" (40.4%), and 27.3% hoped “that someone who couldn’t otherwise get on to biologic treatment would benefit”^28^.

Other data showed that the cost saved by switching patients to biosimilars could enable more efficient allocations of health system resources thus improving patient care. The availability of biosimilars is an opportunity to reduce the price of biological therapies that is the main (and sometimes the only) limiting factor in many countries, as demonstrated in several studies^29–33^ such elements could allow an early access to biological treatments for the patients.

Moreover, the switch is an incentive for the originator pharmaceutical companies not only in reducing prices but also to invest in researching and developing new drugs^21,34^.

The price of biosimilars is 15–75% lower than the originator; since the intrinsic properties of biosimilar drugs (that are not generic drugs but bioequivalent), the interchangeability is a medical decision in almost all countries in Western Europe and in the USA. Therefore, this shift is not to be made by pharmacists or by others in order to prevent an automatic substitution^10,35^.

In literature, several studies and real-world data analysed the short-term impact of the switch from the anti-TNF originator to its biosimilar suggesting that there is a good maintenance of efficacy and safety; however, there are many reports of discontinuation due both lack of efficacy or adverse events. The percentage of interruption varies between 4 and 18%^17,19,36–46^. A Dutch study on 192 patients showed the highest percentage of discontinuation (24%); a sub-analysis of its data verified that the interruption was mainly related to subjective features such as tender joints and patient global assessment rather than objective variables. This phenomenon could be due to a nocebo effect^43,47^ and would require further investigations.

Besides, more recent data from the extension of observation in DANBIO registry confirmed that a certain percentage of switches failed due to patients factors rather than elements related to drug effects^48^.

However, a critical review emphasized the unbalanced cohort and results, asserting that, as of today, there is no study that properly follows FDA guidelines which state that randomised double-blind trials should be included in order to ensure: (a) the homogeneity of treatment groups and control bias; (b) an adequate control with measurement of different outcomes; (c) a proper statistical powering and an appropriate statistical analysis with a well-established evaluation of immunogenicity-related outcomes; (d) an adequate follow-up and assessments of individual patient-level outcomes to support the switch definitively^49,50^.

### Reasons to switch, regional guidelines and aim

In Italy, the Italian competent authority for drugs (AIFA-Agenzia Italiana del Farmaco) published a position papers in 2018 about biosimilars and switching. Although AIFA leaves the final decision to the rheumatologist, it also encourages physicians to strongly consider literature data about the safety and efficacy of the switch, reminding the physicians of their role and responsibility in the economic sustainability of the health system^51^.

In Italy, biological drugs are fully refunded by the health system. After the authorization of the EMA, AIFA issues a decree in order to establish the class and the price of the drug; consequently, the marketing authorisation is granted. As soon as this decree is issued, each Italian region inductees an auction for the award of a public contract between the different producers; the winner of this auction is then permitted to sell their drug in the aforementioned region.

In Piedmont, SB4/Benepali won the auction vs Enbrel in 2017 with a significant price difference.

In our Region, after the AIFA approval for the reimbursement, a commission including members of the Regional Pharmaceutical Service and rheumatologists was established in order to produce a regional guidance on prescribing drugs for naïve patients and in case of switch. The prescription of biosimilars is highly recommended for naïve patients that require a specific target therapy, whilst the switch from originators to biosimilars is encouraged for all the patients treated with the originator; however, some peculiar exceptions are established: patients with history of allergy and/or particularly hypersensitive skin, off-label prescriptions, psychological reasons, active disease that requires a different treatment in the short term, paediatric patients, pregnancy^52,53^.

The regional recommendations refer exclusively to Etanercept and do not preclude in any way the possibility of prescribing the most suitable bDMARD or tsDMARD for the individual patient.

The aim of this work is to evaluate the disease activity trend after switching from etanercept originator Enbrel (oETA) to its biosimilar SB4/Benepali (bETA) in a population admitted to Città della Scienza e della Salute Hospital in Turin, Piedmont, Italy.

Considering that no changes in clinical outcomes were expected, and according to Regional recommendations, we properly discussed about these elements with every patient. In addition, the informed consent was binding in the physician’s final choice as the shared decision between rheumatologists and patients was mandatory^54,55^.

## Materials and methods

We selected patients with clinical diagnosis of Rheumatoid Arthritis (RA), Psoriatic Arthritis (PsA) and Ankylosing Spondylitis (AS) who were admitted to the Rheumatology Unit of the University Hospital of Turin, Italy. The patients had been treated with oETA Enbrel^®^ and switched to bETA Benepali^®^. As suggested by a Regional document, patients off-label, pregnancy and paediatric, patients with history of allergy, patients not in remission nor in low disease activity, patients with psychological reasons that forbid a change were excluded. As per EULAR guidelines^54^, we also excluded patients that refused the switch. At the time of the switch, every patient was informed about biosimilar properties, literature data and the possibility to return to originator if necessary. Almost all patients accepted the switch.

Sample analysis was stratified by age, sex, duration of disease and concomitant therapy. The disease activity was evaluated during the year before the introduction of the bETA, and then the trend of the disease activity was evaluated in the following 12 months during oETA treatment. Patients that stopped therapy for any reason has been evaluated only until the bETA interruption. It was also examined whether some baseline characteristics, such as the duration of treatment with oETA, concomitant therapy (conventional synthetic DMARDs and glucocorticoids) and disease activity, could influence the response to biosimilars.

Statistical analysis was performed using SPSS software (version 17.0, Windows 10 Pro build 1803) and MINI-TAB (version 14.0, Windows 10 Pro build 1803). Descriptive statistics will be provided for the clinical and laboratory demographic characteristics of the cases. In order to evaluate the presence of statistically significant differences between the parameters considered, the χ^2^ test for parametric variables was used. The comparison between groups was performed using the Kruskal–Wallis test and the U-Mann–Whitney test. All the performed tests were bilateral and the level of significance was set at 5% (with a 95% confidence interval).

Multivariable analysis with logistic regression was performed in order to analyze the association of interruption therapy with disease activity, concomitant therapy and oETN duration (age, gender and disease duration were already normalized at the beginning).

## Results

87/107 patients were included (37 male, 50 female) with a median age of 63.0 years old (IQ range 52.2–83.0); the patients were divided by pathology (RA, PsA and AS) while analyzing BMI, ACPA and RF positivity, treatment lines, duration of disease, duration of therapy with oETA and concomitant therapies (csDMARDs) (see Table 1). Patients treated with csDMARDs took Methotrexate in 96% of cases, with a dose between 10 and 15 mg per week. The comparisons of the progress of disease activity were evaluated for the different pathologies (RA, PsA and AS) with their respective clinimetric indices (DAS28, DAPSA, BASDAI, see Table 2). Data analysis showed there are no significant differences in clinimetric parameters after the switch from originator drugs to biosimilars. 11/85 patients (12.6%) stopped the treatment after switch to biosimilar drugs (bETA) due to lack of efficacy (LOE), subjective features (SF) and adverse event (AE); amongst these patients, 5 were affected by RA (3 LOE, 1 SF, 1 AE), 5 patients were affected by PsA (3 LOE, 1 SF, 1 AE), and 1 patient was affected by AS (1 LOE) (Table 3). The AE were not serious: one RA patient showed psoriasis whilst the second one experimented cutaneous rash and diffuse itch. Furthermore, a univariate analysis was performed in order to verify a possible correlation between the interruption of the therapy with bETA and the disease activity at the onset (RA p: 0.231; PsA p: 0.545; AS p: 0.823), the concomitant therapy (RA p: 0.555; PsA p: 0.623; AS p: 0.213) and the duration of oETA treatment (RA p: 0.426; PsA p: 0.676; AS p: 0.522).

**Table 1:** Patient characteristics at baseline.

	Pt (n)	Age (M*, IQ; yy)	Sex (n M/F)	BMI (M*, IQ; yy)	ACPA+ (%)	RF+ (%)	HLA-B27 (%)	Line of treatment (M*, IQ; n)	Duration of disease (M*, IQ; yy)	Duration therapy with bio originator (M*, IQ; yy)	Concomitant csDMARD (%)
RA	48	64 (53–83)	12/36	24.3 (22.6–26.9)	70.8 (N = 34/48)	66.6 (N = 32/48)	–	2 (2–7)	16 (10–38)	7 (3–16)	72,9
PsA	26	63 (57–78)	14/12	26.8 (26.2–28.7)	–	–	–	2 (2–3)	15 (13–28)	7 (3–15)	77,7
AS	13	50 (47–67)	11/2	26.8 (24.8–34.7)	–	–	69.2 (N = 9/13)	3 (2–5)	13 (10–45)	6 (2–12)	15,3

All RA patients has been screened for ACPA and RF while all AS patients has been screened for HLA-B27.

*RA* rheumatoid arthritis, *PsA* psoriatic arthritis, *AS* ankylosing spondylitis, Anti-Citrullinated Protein Antibodies; rheumatoid factor; *oETA* etanercept originator, *csDMARD* conventional synthetic disease modifying anti-rheumatic drug, *BMI* body mass index, M* median, *IQ* inter-quartile range, *M* male, *F* female, *yy* years.

**Table 2:** Correlation between disease activity and drug therapy (oETA and bETA) before switch and after switch, during 6 and 12 months of follow-up.

	oETA Pre-Switch (M*- IQ)	bETA Bsl (M*- IQ)	bETA 6th (M*- IQ)	bETA 12th (M*- IQ)	oETA/bETA Bsl (p-val)	oETA/bETA 6th (p-val)	oETA/bETA 12th (p-val)	bETA Bsl-6th (p-val)	bETA Bsl-12th (p-val)	bETA 6-12th (p-val)
RA (48 pts) DAS28	2.5 (1.8–4.8)	2.5 (1.9–4.7)	2.40 (1.8–7.8)	2.84 (1.8–4.7)	0,740	0,545	0,793	0,742	0,405	0,443
PsA (26 pts) DAPSA	10.0 (6.0–31.0)	7.0 (5.0–24.0)	10.5 (4.0–46.0)	14.9 (6.4–40.8)	0,461	0,506	0,598	1,000	0,205	0,231
AS (13 pts) BASDAI	1.7 (1.3–9.0)	2.0 (1.0–7.5)	2.55 (1.0–8.6)	1.75 (1.0–5.,6)	0,697	0,646	0,596	0,901	0,750	0,525

*RA* rheumatoid arthritis, *PsA* psoriatic arthritis, *AS* ankylosing spondylitis, *DAS28* Disease Activity Score 28, *DAPSA* Disease Activity in Psoriatic Arthritis, *BASDAI* Bath Ankylosing Spondylitis Disease Activity Index, M* median, *IQ* inter-quartile range, *Bsl* baseline, *p-val* p-value.

**Table 3:** Patients that stopped bETA for any reason.

PTS	Sex	Age	DGN	DIS DUR (yy)	oETA (mm)	bETA (mm)	LoE	AE	SF	Measures taken	Outcome after interruption
1	F	68	RA	10	26	4	√	–	–	Swap to a tsDMARD	Clinical improvement
2	F	58	RA	21	34	8	√	–	–	Swap to an other bDMARD	Partially improvement
3	F	85	RA	22	102	6	√	–	–	Swap to an other bDMARD	Clinical improvement
4	M	69	RA	14	92	7	–	Psoriasis (new onset)	–	Switch back to originator	Resolved
5	F	63	RA	16	78	6	–	–	Arthralgia worsening	Switch back to originator	Resolved
6	F	66	PsA	4	26	8	√	–	–	Swich to an other bDMARD; swap to an other bDMARD	Coutaneous rash; clinical improvement
7	F	71	PsA	18	186	6	√	–	–	Swich to an other bDMARD	Partially improvement
8	F	70	PsA	17	52	7	√	–	–	Swich to an other bDMARD	Clinical improvement
9	F	74	PsA	10	89	1	–	Cutaneous rash	–	Switch back to originator	Resolved
10	F	75	PsA	10	45	4	–	–	Arthralgia worsening	Switch back to originator	Resolved
11	M	58	AS	13	84	4	√	–	–	Switch back to originator	Resolved

*PTS* patients, *DGN* diagnosis, *DIS DUR* disease duration, *LoE* lack of efficacy, *AE* adverse event, *SF* subjective features, *yy* years, *mm* months, *bDMARD* biologic disease-modifying antirheumatic drug, *tsDMARD* targeted synthetic disease-modifying antirheumatic drug.

Moreover, we performed the multivariable analysis with logistic regression to verify a possible correlation between the interruption of therapy with bETA, confounders and exposure variables (Table 4).

**Table 4:** Evaluation of variables related to therapy discontinuation at the time of the switch from oETA to bETA.

Variables	Odds ratio	95% CI	p-value
Age	1.185	0.864–1.871	0.093
Gender (M vs F)	0.968	0.598–1.312	0.623
Disease Duration	0.965	0.516–0.133	0.231
Concomitant Therapy	0.996	0.898–1.864	0.213
Duration of oETA treatment (months)	0.879	0.522–1.467	0.676
	1.042	0.783–1.925	0.426
	0.898	0.601–1.142	0.545
	1.070	0.671–1.507	0.823

Disease activity has been assessed with DAS28 for RA patients, DAPSA for PsA and BASDAI for AS on each follow-up visit; there were no missing data.

*RA* rheumatoid arthritis, *PsA* psoriatic arthritis, *AS* ankylosing spondylitis, *DAS28* Disease Activity Score 28, *DAPSA* Disease Activity in Psoriatic Arthritis, *BASDAI* Bath Ankylosing Spondylitis Disease Activity Index, *oETA* etanercept originator, *bETA* etanercept biosimilar, *CI* confidence interval.

## Discussion

Biosimilars drugs are similar to the originator in terms of quality, safety and effectiveness but there are many open questions about ethical implications.

The main concerns are those regarding the switch from the originator to its biosimilar product; the main doubts cover non-medical substitutions which could be performed for situations not related to drug's efficacy nor tolerability nor other medical reasons^10,56^.

The European Medical Agency (EMA) leaves the authority about interchangeability or substitution to each national agency.

Despite the lack of European guidelines, there is an ever-growing practical experience that demonstrates the safety and clinical effectiveness of biosimilars, as well as the savings generated from their introduction in clinical practice^39,57–59^. Therefore, further expensive trials could be avoided to demonstrate an already existing knowledge.

A large difference exists between Western Europe and Eastern Europe. In the latter, access to expensive drugs is limited so the automatic substitution is in some cases allowed, and in many other cases regulated by the law^60^.

The Italian competent authority for drugs (AIFA-Agenzia Italiana del Farmaco) published two position papers about biosimilars and switching. The aim of the documents was to provide health professionals and patients clear and validated information about biosimilars, including the role of biosimilars in the economic sustainability of the National Health Service. Even if the final decision about the switch is entrusted to the physician (after a proper informed consent given by patients) AIFA sustains the interchangeability of biosimilars and emphasizes the physician role in economic sustainability of the health system^52^.

The analysis of our real-life data in those patients who agreed to switch, confirms what has already emerged from clinical trials and real-world data in the literature. In particular, there were no statistically significant differences in disease activity after the switch to bETA and during the follow up (1 year after the switch). Furthermore, no correlations emerged between the interruption of treatment with bETA and the variables analysed. The adverse events were not serious. Amongst real-life data reports^61–67^, we collected data for up to 12 months of follow-up. However, this descriptive study has some limitations since it includes no data about pharmacokinetics or evaluation of anti-drug antibodies, from neither the originator nor the biosimilar. Finally, the nocebo effect was not investigated with psychometric measures.

In addition to this, 20 patients were excluded (21.4% of the sample); despite this element may seem to limit the study, it should be taken into consideration that 12 of these patients were carrying out therapies as per off label dose reductions (thus not being comparable with the rest), whilst 4 had psychological reasons (which are likely to be comprised inside the Nocebo effect) and 4 of them were paediatric patients. So, it can be concluded that there is an equal balance of negative and positive aspects that make this data loss less significant.

In conclusion, in our population, no difference has been observed with regard to efficacy and safety after the switch from originator to biosimilar, and no predictors of non-response to switch therapy are currently highlighted. In our opinion, the switch could be considered safe. The physician–patient cooperation play a key role for a successful switch; therefore, it is critical to ensure that patients are informed about all the relevant information related to the switch as well as the respect for patients decisions and to make a strict follow-up to check any AE immediately.

It is also fundamental to share clinical reports to improve real life data.
